# Spectacles with highly aspherical lenslets for myopia control do not change visual sensitivity in automated static perimetry

**DOI:** 10.3389/fnins.2022.996908

**Published:** 2022-11-25

**Authors:** Yi Gao, Daniel P. Spiegel, Izzah Al Ilma Muzahid, Ee Woon Lim, Björn Drobe

**Affiliations:** Essilor R&D Centre, Singapore, Singapore

**Keywords:** myopia, myopia control, perimetry, visual sensitivity, highly aspherical lenslets (HAL), visual field, spectacles

## Abstract

**Purpose:**

Spectacle lenses with arrays of lenslets have gained popularity in myopia control due to their high efficacy, low impact on visual performance, and non-invasiveness. One of the questions regarding their impact on visual performance that still remain is that: do the lenslets impact visual field sensitivity? The current study aims to investigate the impact of wearing spectacle lenses with highly aspherical lenslets (HAL) on the visual field sensitivity.

**Methods:**

An automated static perimetry test (Goldman perimeter target III) was employed to measure the detection sensitivity in the visual field. Targets were white light dots of various luminance levels and size 0.43°, randomly appearing at 76 locations within 30° eccentricity. Twenty-one adult subjects (age 23–61, spherical equivalent refractive error (SER) −8.75 D to +0.88 D) participated in the study. Sensitivities through two lenses, HAL and a single vision lens (SVL) as the control condition, were measured in random order.

**Results:**

The mean sensitivity differences between HAL and SVL across the 76 tested locations ranged between −1.14 decibels (dB) and 1.28 dB. Only one location at 30° in the temporal visual field reached statistical significance (*p* < 0.00065) whereby the sensitivity increased by 1.1 dB with HAL. No significant correlation was found between the difference in sensitivity and age or SER. Such a difference is unlikely to be clinically relevant.

**Conclusion:**

Compared to the SVL, the HAL did not change detection sensitivity to static targets in the whole visual field within 30° eccentricity.

## Introduction

The prevalence of myopia is growing around the globe (Fricke et al., 2018). Early administration of myopia control is needed to reduce the high risk of ocular pathologies due to high myopia (Wong et al., 2018). A number of myopia control interventions include light therapy (Jiang et al., 2022; Zhou et al., 2022), orthokeratology (Cho and Cheung, 2012; Cho and Tan, 2019; Loertscher et al., 2021), soft contact lenses (Anstice and Phillips, 2011; Chamberlain et al., 2019), and spectacle lenses (Lam et al., 2020b; Bao et al., 2021, 2022). In particular, spectacle lenses represent a very attractive myopia control solution because they are non-invasive and safe to wear. Among all myopia control spectacle lenses, lenses with arrays of lenslets emerge as particularly effective in controlling myopia progression (Lam et al., 2020b; Bao et al., 2022). The 2 year clinical trial results of spectacles lenses with highly aspherical lenslets (HAL) showed that the lens slowed down myopia progression by 0.80 D in spherical equivalent refraction (SER), and 0.35 mm in axial length elongation compared with single vision lens (SVL) (Bao et al., 2022). The clinical trial on the Defocus Incorporated Multiple Segments’ (DIMS) spectacle lens for the same duration showed a myopia control efficacy of 0.55 D in SER, and 0.32 mm in axial length elongation for children in the DIMS group compared with those in the SVL group (Lam et al., 2020b).

In previous studies (Gao et al., 2021; Li et al., 2021), the HAL lens was found to have only a small impact on visual acuity. When looking directly through the lenslets, high contrast VA was reduced by HAL for 0.07 logMAR, which is only slightly more than half a line, in children (Li et al., 2021); for adults (Gao et al., 2021), high contrast VA was also reduced by 0.07 logMAR and low contrast VA was reduced by 0.14 logMAR. Another myopia control spectacle lens with a honeycomb configuration of spherical lenslets (HC) was found to reduce VA for about one line (Li et al., 2021). In the same study, contrast sensitivities (CS) at various conditions were also tested through the lenslets areas of these lenses. It was found that HAL reduced CS slightly mainly at mid-high spatial frequencies (SF), especially at a low luminance level and in conditions with added glare. HC was found to influence CS to a larger degree in various conditions (Li et al., 2021). The study conducted on adults (Gao et al., 2021) found that HAL had no significant impact on peripheral visual functions including useful field of view (UFOV), peripheral motion detection, and peripheral coherent motion direction discrimination.

In particular, UFOV, which is a clinical tool used in driving license screening (Wood and Owsley, 2014) to test the divided attention in the visual field was used to characterize the HAL lens. The test presents the peripheral target at one of the eight concentric locations randomly with and without distractors and measures the shortest display time for the target to be detected. It remains to be determined whether wearing HAL has an impact on the detection CS at different locations across the visual field. To address this question in this study, static automated perimetry, which measures the detection luminance thresholds of static targets at various locations in the visual field, was tested through a HAL lens and a SVL. In line with previous findings, we hypothesized that there was no clinically significant reduction in sensitivity across the visual field.

## Method

### Subjects

The inclusion criteria were age 21–65, refractive error of sphere between −10 and +10 D, and astigmatism not more than 1 D. People with any ocular pathologies or history of them were excluded. Participants were recruited through an internal subject database and word-of-mouth. Twenty-one participants volunteered in this study. Their age ranged from 23 to 61 (mean = 38.1 ± 10.4) years, and the spherical equivalent refractive errors (SER) ranged from −8.75 to +0.88 D (mean = −2.76 ± 2.83). The right eye was used for testing while the left eye was patched with an opaque eye patch. The subject’s information was entered into the machine to calculate the compensated prescription for the short viewing distance. Signatures for the informed consent forms were obtained from all participants prior to any procedure of the study. The study was conducted in accordance with the Helsinki Declaration of Principles and approved by the Parkway Independent Ethics Committee in Singapore (#PIEC/2021/020).

### Study lenses

The test lens was a plano HAL. The lens contains 11 concentric rings of contiguous aspherical lenslets centered around a 9 mm-diameter clear zone. More details of the lens design have been provided in the article of 1 year clinical trial results (Bao et al., 2021). It was edged into a 36.5 mm trial lens ring to fit the lens holder of the perimeter. The HAL was positioned to have the central clear zone of 9 mm diameter centered on the geometric center of the trial lens ring. Six out of the 11 concentric rings of contiguous aspherical lenslets of the unedged lens remained in the peripheral zone after edging. Study lenses were fitted into the lens holder of the machine in front of the trial lenses with compensated prescription of each subject. The compensated prescription was calculated based on the viewing distance of 30 cm, each subject’s prescription, and the age of the subject. The machine has an embedded algorithm to perform the calculation and to display the result. It was then delivered using the trial lens set supplied with the perimeter. The control lens was the plano SVL from the trial lens set. During the experiment, the lenses were aligned in front of the subjects’ right eye and the perimetry test was performed by looking through the central area of the lenses. The testing order of the two lenses was counterbalanced and pre-allocated for all subjects. The order was unknown to the subjects.

### Test and procedure

The automated static visual field test “30-2 threshold” on an Optical Kinetic Perimeter SK-950B (Chongqing Sunkingdom Medical Instrument Co., Ltd, China) was used. The test measures sensitivity to static white light dots within 30° from an orange fixation light. In total, 76 locations of the visual field were tested. Targets of white light dots, Goldman III, size 0.43°, of different luminance levels appeared for 0.2 s, at random locations in the tested field. The subjects were instructed to press a click button whenever they saw a target, while continuously fixating on the central fixation light. The viewing distance was 30 cm. Throughout the test the machine tracked the pupil position and automatically adjusted the chinrest to ensure correct eccentricities. The machine also monitors fixation. A practice run was done without any lens to ensure that the subjects were familiar and comfortable with the task. Each session lasted about 4–5 min. The software of the machine deems a run unreliable if fixation loss is more than 20%, false negative errors are more than 20%, or false positive errors are more than 15%. Then the run was repeated. At the end of each test, results were saved and stored in the machine, before exporting it for data analysis.

### Analysis

The data were tested for normality using Kolmogorov–Smirnov test at each retinal location. Many locations showed significant difference from normal distribution. Therefore, non-parametric tests were used for further analysis. To quantify the difference between the SVL and the HAL lens in visual sensitivity across the visual field, we compared the raw sensitivity data obtained through the two lenses using the Wilcoxon signed rank test conducted individually for each retinal location. Bonferroni correction for multiple comparisons was applied to avoid a type I error (Armstrong, 2014). It required adopting a significance level of *p* < 0.00065. Effect size was also calculated. All analysis was conducted using MATLAB R2020b (The MathWorks Inc., Natick, MA, USA). (MATLAB, 2020).

## Results

**FIGURE 1:**
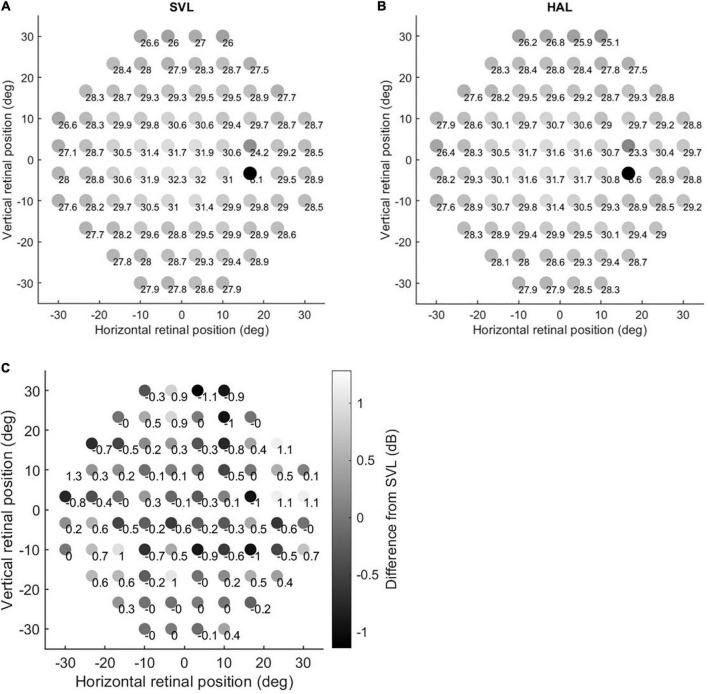
Sensitivity maps. Panels **(A,B)** show the raw sensitivity data through the single vision lens (SVL) and the highly aspherical lenslets (HAL), respectively. The grayscale is proportional to the mean values. Panel **(C)** shows the mean difference between values in Panels **(B,A)**. Accordingly, positive numbers indicate increased sensitivity through the HAL and negative numbers indicate decreased sensitivity through the HAL compared to the SVL.

The average raw sensitivity data across subjects for each lens are shown in Figure 1 (A for SVL and B for HAL). The average differences between the two lenses are depicted in Figure 1C. The differences (mean ± SD) ranged between −1.14 ± 3.07 and 1.29 ± 3.84 decibels (dB) and the *p*-values (Wilcoxon signed rank test) ranged from 0.0005 to 0.97. Based on our significance level (*p* < 0.00065) accounting for multiple comparisons, only one location at 30° in the temporal visual field (Row 5, Column 10 in Figure 1C) showed a significant difference (*p* = 0.0005, effect size = 0.4) in sensitivity between the HAL and the SVL lenses. Specifically, the sensitivity at that retinal location was on average 1.1 dB higher with the HAL than the SVL. Considering the fact that no other (neighboring) retinal location showed a significant difference and the small effect size of the difference, this result is unlikely clinically meaningful. There was no significant correlation (Spearman’s rank correlation) between the difference in sensitivity at that specific location with the age (*r* = 0.11, *p* = 0.62) or the SER (*r* = −0.002, *p* = 0.99) of the subjects.

## Discussion

In this study, we found no evidence of reduced sensitivity across the visual field associated with the highly aspherical lenslets using automated static perimetry.

This is the first study using perimetry to evaluate possible changes in visual sensitivity associated with wearing myopia control spectacle lenses with aspherical lenslets. In fact, not many studies have evaluated potential changes in visual sensitivity with specially designed spectacle lenses or with “simple” optical defocus using perimetry. An early report showed that positive optical defocus of 1.5 and 4 D reduced CS in static perimetry in the visual field up to 30° for small targets that were equal or smaller than Goldmann perimeter target III (26 min arc diameter) (Atchison, 1987). Broadly in agreement was a later study showing significant decrease in detection sensitivity for small perimetry stimuli (equal or smaller than 0.8°) with increasing peripheral defocus up to 4 D (Anderson et al., 2001). On the other hand, optical defocus up to 6 D had only little effect on visual sensitivity in frequency-doubling perimetry (Anderson and Johnson, 2003). A more recent study applied automatic kinetic perimetry and measured the effect of positive optical defocus of 0–7 D induced by soft contact lenses. Only the kinetic sensitivities of low-intensity stimuli were slightly affected by the larger amount of defocus (Hirasawa and Shoji, 2015). In summary, the results of the previous studies investigating the effects of optical defocus on the perimetry measurements were equivocal mainly due to the small number of studies with various methodologies. Due to the differences in our and previous methodologies, it is difficult to compare their findings. Overall, we found no evidence of an impact of the HAL on detection sensitivity in central and peripheral vision using small static stimuli (smaller than 1°). Although some visual functions such as visual acuity and CS can be sensitive to defocus, we speculate that our finding of no impact is due to the nature of peripheral vision being coarser, and the design of HAL where the lenslets cover only 40% of the lens area outside of the central clear zone. Therefore, 60% of the light passing through HAL remains focused on the retina rendering normal visual performance. The patterns of defocus were also different between the current and the previous studies. In the current study, concentric rings of aspherical lenslets in the area of higher than 18° eccentricity create non-continuous defocus volume in front of the retinal; while in previous studies, fixed and uniform amounts of defocus for the whole lens were adopted.

A number of studies have evaluated the impact of HAL on other aspects of visual perception including central visual acuity (Gao et al., 2021; Li et al., 2021), central CS (Li et al., 2021), peripheral motion perception and UFOV (Gao et al., 2021). The impact of lenslets on central CS was found to be small or non-existing. For example, the central contrast threshold at six cycles per degree through the HAL was increased by less than 0.5% compared to the SVL (Li et al., 2021). At the same spatial frequency, the contrast threshold increased linearly with eccentricity, from about 0.8% at zero degree to about 32% at 12° (Wright and Johnston, 1983). The contrast threshold at larger eccentricities would be even higher. It is not surprising that a difference of 0.5% (Li et al., 2021) did not affect peripheral vision. The impact on central visual acuity through the lenslets was also found to be 0.07 logMAR, less than one line (Gao et al., 2021; Li et al., 2021). The impact of the HAL on all peripheral visual tasks such as peripheral motion detection, cardinal motion and UFOV (Gao et al., 2021) was also found to be not significant. In summary, the finding of no negative impact on visual sensitivity in the visual field within 30° using automated static perimetry is consistent with previous findings that the wearing of the HAL exhibited no significant impact on peripheral vision.

The ability to detect objects in different parts of the visual field is important for road safety and visual field tests have been used in drivers’ license screening in some countries, for example, see UK Driver and Vehicle Licensing Authority guidelines. Good visual sensitivity in the whole visual field is also needed for sports performance (Popowczak et al., 2020) and reducing risks of sports injuries (Kung et al., 2020). Although only a static detection test was performed in the current study, the results suggest that the possibility that road safety or sports performance would be hindered by wearing HAL is reasonably low.

Note that in this study, luminance sensitivity of white dots having energy in the full spatial frequency range was measured. An earlier study found that central CS through the lenslets was affected at only mid to high spatial frequencies (6–18 cycles per degree), while low SF CS was not affected (Li et al., 2021). Considering visual acuity in the periphery at further than 18° was as low as 1.0 logMAR (Randall et al., 1966; Frisén and Glansholm, 1975), testing mid to high spatial frequency would be quite impossible. Therefore, we chose not to test various SFs separately. Instead, we measured the luminance sensitivity with a clinically available test for easier interpretation by eye care practitioners. Our finding indicates that wearer’s ability to detect low-contrast and small objects is not affected. Considering the characteristics of peripheral vision, detecting higher-contrast and bigger targets in wearers’ daily life should not be affected either.

Our study has some limitations. First, we tested the lenses in an adult population and in some cases in non-myopes whereas wearers of HAL were mainly myopic children. Visual performance may differ between children and adults (Friendly, 1993; Braddick and Atkinson, 2011), however, the impact of spectacle lens design on vision can be similar. For example, two former studies that tested the impact of the HAL on far visual acuity at 100% contrast found a similar reduction of 0.07 logMAR in both children (Li et al., 2021) and adults (Gao et al., 2021). Thus, we can speculate that our findings can be generalized to the children population. Also, although the impact of HAL on visual field sensitivity in the periphery can be different between myopes and non-myopes due to the difference in their eye shapes, any potential impact caused by the lenslets in the periphery should be smaller in myopes than non-myopes. Because in normal circumstances with correction of regular SVL, in the peripheral visual field the myopic eyes have hyperopic defocus while the hyperopic eyes have myopic defocus. With the HAL lens, the plus power of the lenslets compensates the hyperopic defocus and causes myopic defocus for the myopic eyes, while enlarges the myopic defocus for the hyperopic eyes. Therefore, there is smaller myopic defocus on the peripheral retina in the myopic eyes than non-myopic eyes. Hence, even if the HAL lens reduced peripheral visual field sensitivity, the effect would be smaller in myopes than in non-myopes. Since we found no reduction in visual field sensitivity by the HAL lens in our participants with various forms of ametropia, our results suggest that the HAL lens would not reduce visual field sensitivity if the participants were all myopes. Further studies on myopic children with a larger sample size can provide more direct and relevant findings.

Second, we used a static test instead of a dynamic test. In terms of being relevant to daily activities of the wearers, a kinetic test could be more appropriate. However, the kinetic test cannot be run with the lens holder in the upward position due to the small size of the testing lens ring. Also, the size of the testing ring is smaller than the range of the kinetic test. Therefore, we could not test the impact of the HAL lens on the kinetic test. Future research measuring the impact of HAL on the kinetic sensitivity in the visual field would provide further valuable insights. The short viewing distance of 30 cm and the range of 30° of the perimetry tests also limit the scope in which our findings can apply to real-world scenario. The experimental result of no reduction in sensitivity may not generalize widely to activities in the daily life that require far and wide peripheral vision. Further studies are needed to assess potential impact at further distances and higher eccentricities.

Third, we only tested short-term effect, i.e., our participants were exposed to the HAL only in the lab during testing. Usually after long-term adaptation, visual functions through lenses of special designs do not deteriorate. For example, Lam et al. (2020a) found that after 2 years of wearing, far high-contrast VA through the DIMS lens, as well as the SVL, improved compared to the baseline; while far low-contrast VA and near high- and low-contrast VA did not change. It is also common to conduct visual tests on short-term effects. Future work where myopia control spectacle lenses are adapted and worn for at least 6 months will provide more insight into the long-term effects of these lenses on visual perception.

## Data availability statement

The raw data supporting the conclusions of this article will be made available by the authors, without undue reservation.

## Ethics statement

The studies involving human participants were reviewed and approved by Parkway Independent Ethics Committee, Singapore. The patients/participants provided their written informed consent to participate in this study.

## Author contributions

YG, DS, and BD contributed to the conception and design of the study and to the manuscript revision. YG set up the experiment. IM, DS, and EL performed the data collection. IM organized the database. DS performed the data analysis and figure plotting. YG and DS wrote the first draft of the manuscript. IM and EL wrote “Method” sections of the manuscript. All authors read and approved the submitted version.
